# Supplementary material for: Schizophrenic patients with type 2 diabetes: An 8-year population-based observational study
Source: PLoS One. 2021 Mar 16;16(3):e0248407. doi: 10.1371/journal.pone.0248407 (PMC7963070; doi:10.1371/journal.pone.0248407)
Supplement: S1 File — (PDF) [file pone.0248407.s001.pdf]

Results: A total of 6.8% (87/1287) of persons died of natural causes. There were 70 deaths in the schizophrenia and 17 in the bipolar disorder participants. The mean age at death of those who died from natural causes was 56.7 years (range 19.4 – 79.1 years). The standardized mortality ratio (SMR), the age-adjusted ratio of the number of observed deaths in this study sample to that expected in the general population, was 2.57 (95% CI 1.24 – 4.75).

Natural cause mortality was predicted in a multivariate model by baseline cigarette smoking (RR=6.29, 95% CI 1.41, 3.72, p=0.00076); divorced or widowed status (RR=1.90, CI 1.21, 2.99); reduced cognitive score (RR=0.73, CI 0.61, 0.87); receipt of antidepressant medication (RR=1.74, CI 1.12, 2.71); elevated levels of antibodies to Epstein Barr Virus (EBV) (RR=1.29, CI 1.01, 1.66); and a genitourinary (RR 1.82, CI 1.16, 2.86), respiratory (RR 1.82, CI 1.16, 2.86), or cardiac (RR 2.09, CI 1.33, 3.29) condition.

Interaction models showed evidence of additive effects of smoking and both cardiac and respiratory condition. Compared to non-smokers without a cardiac condition, non-smokers with a cardiac condition had a more than threefold elevation of mortality risk (RR=3.76, 95% CI 1.47 – 9.63, p=0.0057) as did smokers without a cardiac condition (RR=3.63, 95% CI 1.49 – 8.85, p=0.0046), while the presence of both smoking and a cardiac condition increased mortality risk by more than six-fold (RR=6.75, 95% CI 2.84 – 16.0, p<0.0001). Compared to non-smokers without a respiratory condition, mortality risk more than doubled for non-smokers with a respiratory condition (RR=2.30, 95% CI 0.97 – 5.46, p=0.058), as well as for smokers without a respiratory condition (RR=2.37, 95% CI 1.31 – 4.28, p=0.0044), while the mortality risk more than quadrupled for smokers with a respiratory condition (RR=4.72, 95% CI 2.45 – 9.09, p<0.0001). There was not a significant interaction between smoking and elevated EBV antibody levels. There was a synergistic effect of antidepressant use and cardiac disease on mortality risk: participants with both risk factors had a more than threefold elevation of mortality risk compared to persons with neither risk factor (RR=3.10, 95% CI 1.71 – 5.63, p=0.0002).

Discussion: Multiple factors contribute to the excess mortality of persons with schizophrenia and bipolar disorder, but cigarette smoking is a major preventative cause. The delivery of smoking cessation treatments should be a high priority.

O5.5. SLEEP IN MAJOR PSYCHIATRIC DISORDERS: RESULTS FROM NATIONWIDE SUPER FINLAND STUDY

Minna Torniainen-Holm*[,1], Erik Cederlöf[1], Willehard Haaki[2], Jarmo Hietala[2], Katja Häkkinen[3], Erkki Isometsä[4], Kaisla Joutsenniemi[4], Tuomas Jukuri[5], Risto Kajanne[3], Olli Kampman[6], Tuula Kieseppä[4], Nina Lindberg[4], Markku Lähteenvuo[7], Jouko Lönnqvist[1], Teemu Männynsalo[8], Jussi Niemi-Pynttäri[9], Kimmo Suokas[9], Jaana Suvisaari[1], Jari Tiihonen[10], Annamari Tuulio-Henriksson[11], Asko Wegelius[12], Juha Veijola[5], Aarno Palotie[3], Tiina Paunio[1]

[1]National Institute for Health and Welfare, University of Helsinki; [2]University of Turku; [3]Institute for Molecular Medicine Finland, Niuvanniemi Hospital; [4]Helsinki University Hospital, University of Helsinki; [5]University of Oulu; [6]University of Tampere, Seinäjoki Hospital District; [7]Institute for Molecular Medicine Finland, University of Tampere; [8]City of Helsinki, Social Services and Health Care Sector; [9]University of Tampere; [10]Niuvanniemi Hospital, University of Eastern Finland, Karolinska Institutet; [11]University of Helsinki, The Social Insurance Institution of Finland; [12]Helsinki University Hospital

Background: People with psychotic disorders demonstrate a wide spectrum of sleep abnormalities. Abnormalities include changes in total sleep time, increased sleep onset latency, increased wake-up time after sleep onset and abnormalities in sleep architecture. The study aimed to characterize the sleep difficulties in a large sample of persons with psychotic disorders and to examine association with age and gender.

Methods: Altogether, 5046 persons with a major psychiatric disorder (schizophrenia: 2972, schizoaffective disorder: 640, bipolar disorder: 1097 and psychotic depression: 330) and aged 18–80 participated in a nationwide Super project. The Finnish SUPER (Finnish acronym for "Finnish study on genetic mechanisms of psychotic disorders") study is a part of the international Stanley Global Neuropsychiatric Genomics Initiative. The results were compared with a representative general population sample of 8018 adults (Health 2000). Sleep was assessed in a self-report questionnaire. In the present study we used total sleep time, tiredness (defined as feeling more tired than other people of the same age during day time at least weakly, yes/no), difficulties in getting sleep without sleep medication often or almost daily (yes/no) and early morning or night awakenings occurring either often or nearly every night (yes/no).

Results: Long sleep (> 10h) was most common in persons with schizophrenia or schizoaffective disorder reported by approximately 30% when age was 18–40. The corresponding proportion was approximately 15% in persons with bipolar disorder or psychotic depression and less than 1% in the general population. Tiredness, difficulties in getting sleep and early morning or night awakenings were reported most by persons with bipolar disorder and psychotic depression, but also persons with schizophrenia reported those more than the general population in people with under 60 years of age. Schizoaffective disorder was between schizophrenia and affective psychoses in the sleep variables. In persons over 60, the difference between the groups was smaller than in persons under 60 years of age, because sleeping long and tiredness decreased in all patient groups, and difficulties getting sleep and awakenings increased in the general population sample more than in psychosis patients.

Discussion: Sleep disorders seem to be prominent in persons with major psychiatric disorders. Tiredness was common in all diagnosis groups. Long sleep was most common in schizophrenia and difficulties in getting sleep and early morning or night awakenings in affective psychoses. More research is needed on possibilities to prevent and treat sleep disorders in major psychiatric disorders.

O5.6. SUBMISSION WITHDRAWN

O5.7. RISK OF DIABETIC COMPLICATIONS AND SUBSEQUENT MORTALITY AMONG INDIVIDUALS WITH SCHIZOPHRENIA AND DIABETES MELLITUS: A NATIONWIDE POPULATION-BASED REGISTER STUDY

Anita Tønder Nielsen*[,1], Mogens Vestergaard[2], Trine Munk-Olsen[3], Jette Kolding Kristensen[4], Thomas Munk Laursen[5]

[1]The National Centre for Register-based Research, Aarhus University; [2]Institute of General Medical Practice, Aarhus University; [3]National Centre for Register-Based Research, Aarhus University, The Lundbeck Foundation Initiative for Integrative Psychiatric Research (iPSYCH); [4]Research Unit for General Practice, Aalborg University; [5]Aarhus University, The Lundbeck Foundation Initiative for Integrative Psychiatric Research (iPSYCH)

Background: Schizophrenia constitutes a high risk of morbidity and mortality from physical illness. Individuals with comorbid schizophrenia and diabetes mellitus have been found to have a three- to four-fold higher rate of death than the general population, which may be explained by a higher rate

of diabetic complications. We aimed to study incidence of diabetic complications diagnosed in hospitals following a diabetes diagnosis and subsequent mortality in individuals with schizophrenia compared to individuals without.

Methods: The entire Danish population was followed in 1997–2016 using population-based registries. Incident diabetes was defined as prescription redemptions of insulin or oral antidiabetic drugs, ICD-10 diagnoses E10 or E11 related to hospital contacts, whichever came first. Diabetic complications were separated into macrovascular complications (coronary heart disease, peripheral artery disease, stroke+TCI, heart failure, myocardial infraction, foot ulcer) and microvascular complications (retinopathy, neuropathy, nephropathy).

Cox regression was used to estimate incidence rate ratios (IRR) and mortality rate ratios (MRR) and all estimates were adjusted for age and calendar time.

Results: The incidence rate of macrovascular complications was similar in individuals with schizophrenia and in those without; IRR=1.09 (95% CI: 0.96–1.23) for females and IRR=0.91 (95% CI: 0.83–1.01) for males. For foot ulcer the incidence rate was higher in females with schizophrenia than in females without; IRR=1.79 (95% CI: 1.12–2.85), p=0.015 and for heart failure the incidence rate was higher in males with schizophrenia than in males without; IRR=1.52 (95% CI: 1.21–1.91), p<0.000. The incidence rate for microvascular complications was similar for females with and without schizophrenia; IRR=0.88 (95% CI: 0.75–1.04), but lower in males with schizophrenia than in males without schizophrenia; IRR=0.79 (95% CI: 0.69–0.89), P<0.001.

The mortality rate following a diagnosis of a macrovascular complication was higher in individuals with schizophrenia than those without; MRR=2.17 (95% CI: 1.84–2.56) for females and MRR=2.40 (95% CI: 2.06–2.80) for males. For microvascular complications the subsequent mortality rate was also higher in individuals with schizophrenia than in those without; MRR=2.17 (95% CI: 1.67–2.80) for females and MRR=2.30 (95% CI: 1.90–2.79) for males. P<0.001 for all estimates. The Mortality rates for every single complication showed similar estimates.

Discussion: Unexpectedly, we found individuals with comorbid schizophrenia and diabetes mellitus to have a similar or lower rate of diabetic complications diagnosed in hospitals compared to individuals with diabetes mellitus only. However, we still found an excess mortality following a diagnosis of a diabetic complication among individuals with schizophrenia. These results may indicate that individuals are not even seen in hospitals with their diabetic complications and hence indicate an increased need for improved somatic care of individuals with schizophrenia if the burden of diabetes mellitus morbidity and mortality should be reduced.

O6. Oral Session: Neuroimaging

O6.1. HIPPOCAMPAL VOLUME IN ADOLESCENTS WITH PERSISTENT PSYCHOTIC EXPERIENCES: A LONGITUDINAL POPULATION-BASED MRI STUDY

Ana Calvo*[,1], Erik O'Hanlon[2], Helen Coughlan[2], Ian Kelleher[2], Mary Clarke[2], Mary Cannon[2]

[1]Universidad Internacional de La Rioja; [2]Royal College of Surgeons in Ireland

Background: Individuals with schizophrenia show significant brain morphological abnormalities. The ENIGMA consortium identified that patients with schizophrenia had smaller hippocampus, amygdala, thalamus, accumbens and intracranial volumes.[1] Reduced hippocampal volume is one of the most consistent findings in schizophrenia research.[2–4] Also, Previous research has reported differences in hippocampal volume and white matter integrity in young adolescents who report psychotic experiences.[5,6] However there has been little longitudinal research to investigate the developmental trajectory of these regions in adolescence with an increased susceptibility to psychotic disorders.

Aims: to investigate two-year longitudinal changes in hippocampal volume in a sample of adolescents who reported psychotic experiences relative to their peers. To investigate the role of presence of co-morbid DSM IV mental disorders and stressful life events in influencing hippocampal volume and study the differences in hippocampus volume between adolescents who were having persistent symptoms versus adolescents with remitting symptoms.

Methods: A longitudinal case-control study of 50 community-based adolescents aged 13–16 years (25 with psychotic experiences and a matched sample of 25 without psychotic experiences), compared hippocampal volume. All participants were assessed at baseline and two years follow up. T1 weighted anatomical high-resolution imaging and high angular resolution diffusion imaging data were used to conduct quantitative anatomical volumetric evaluations of global hippocampal volume. Clinical interviews also provided information on psychotic experiences, co-morbid disorders and adverse life events.

Results: There were significant differences in the Right and Left Whole hippocampus between PE and Control group at baseline and 2-year follow up (p≤ 0.05). There were significant differences between PE persist and Control group in the left and right whole hippocampus (p≤ 0.05).

Discussion: The differences identified in our study suggest that early hippocampal reductions, may play a role in increasing vulnerability to psychosis.

References:

1. van Erp TG, Hibar DP, Rasmussen JM, et al. Subcortical brain volume abnormalities in 2028 individuals with schizophrenia and 2540 healthy controls via the ENIGMA consortium. Mol Psychiatry. 2016;21(4):585.
2. Wright IC, Rabe-Hesketh S, Woodruff PW, David AS, Murray RM, Bullmore ET. Meta-analysis of regional brain volumes in schizophrenia. Am J Psychiatry. 2000;157(1):16–25.
3. Nelson MD, Saykin AJ, Flashman LA, Riordan HJ. Hippocampal volume reduction in schizophrenia as assessed by magnetic resonance imaging: a meta-analytic study. Arch Gen Psychiatry. 1998;55(5):433–440.
4. Stein JL, Medland SE, Vasquez AA, et al. Identification of common variants associated with human hippocampal and intracranial volumes. Nat Genet. 2012;44(5):552–561.
5. Drakesmith M, Caeyenberghs K, Dutt A, et al. Schizophrenia-like topological changes in the structural connectome of individuals with subclinical psychotic experiences. Hum Brain Mapp. 2015;36(7):2629–2643.
6. Satterthwaite TD, Wolf DH, Calkins ME, et al. Structural Brain Abnormalities in Youth With Psychosis Spectrum Symptoms. JAMA psychiatry. 2016;73(5):515–524.

O6.2. NEUROBIOLOGY OF PSYCHOMETRIC SCHIZOTYPY: INSIGHTS FROM MULTIMODAL IMAGING RESEARCH

Gemma Modinos*[,1], Alice Egerton[1], David J. Lythgoe[1], Gareth J. Barker[1], Katrina McMullen[1], Christian Keysers[2], André Aleman[3], Veena Kumari[1], Steven CR Williams[1]

[1]Institute of Psychiatry, Psychology & Neuroscience, King's College London; [2]Netherlands Institute for Neuroscience, Netherlands Academy for Arts and Sciences, KNAW, University of Amsterdam; [3]University of Groningen, University Medical Hospital Groningen

Background: The continuum approach to psychosis proposes a dimensional continuity between the neurobiology of subclinical psychotic-like experiences in healthy individuals (or schizotypy) and psychotic symptoms in clinically relevant psychosis (Linscott and van Os, 2013, Nelson et al., 2013). Preclinical models propose that cortical glutamate dysfunction related to cortico-limbic-striatal hyper-responsivity to stress may underlie both hippocampal and striatal overdrive as well as gray matter loss associated with schizophrenia-like behaviors (Berretta et al., 2001, Lodge and
