# Supplementary material for: Schizophrenic patients with type 2 diabetes: An 8-year population-based observational study
Source: PLoS One. 2021 Mar 16;16(3):e0248407. doi: 10.1371/journal.pone.0248407 (PMC7963070; doi:10.1371/journal.pone.0248407)
Supplement: Attachment — Submitted filename: Response to Reviewers 1.docx [file pone.0248407.s004.docx]

**Dear Editor of PLOS ONE**

Thank you very much for all your comments and the opportunity to improve the manuscript. We have been working to introduce the comments provided by Reviewers. We hope that the present version of the manuscript will meet the expectations of the Reviewers and Editor of the PLOS ONE. The changes within the revised manuscript were highlighted (underlined and in yellow). Once again, thank you for your valuable comments and attention given to our paper.

Best regards,
Mariusz Jaworski

|  | Reviewer (#R) Comments | **Authors' Response** (#A) |
| --- | --- | --- |
| R#1 | #R  In general, the authors have been responsive to my questions of review. The results are novel and interesting and the methodology seems correct. Overall, I think it is worthy of publication and will be of interest to readers. | #A  We would like to thank the reviewer for the positive review and recommendation of our article for publication in the PLOS ONE journal. |
| R#2 | #R  How the data was analyzed. Which test was used to explain your hypothesis?? | #A  As the aggregated data was provided by the National Health Fund, only descriptive statistics could be presented. However, to estimate the population value of the relative risk (RR), interval estimation was used as a method of statistical inference. For the point estimator (RR) a 95% confidence interval was calculated. Due to the nature of the collected data, the null hypothesis significance test was not used |
| R#2 | #R  Lots of grammatical and typo mistakes e.g., In this paper, we wanted to determine if T2DM is present in every sub-type of schizophrenia??  Is the manuscript presented in an intelligible fashion and written in standard English? No | #A  Thanks for this comment. Our article has been edited by a native speaker of English who is a Doctor of Medicine. I am attaching the certificate. We hope that this version of our article is linguistically much better. |
| R#2 | #R  Has the statistical analysis been performed appropriately and rigorously? No | #A  We have added information on the rules for calculating relative risk. Additional data that was used were also explained. Please see the modified and completed text:  Since the aggregated data were provided by the NFZ, only descriptive statistics were possible to present. The demographic characteristics of the participants were analysed through descriptive statistics. The annual prevalence of schizophrenia was estimated according to the T2DM diagnosis status, and the age groups were organised into eight sets (11–20, 21–30, 31–40, 41–50, 51–60, 61–70, 71 80 and ≥81). The eight sets were defined by the authors on the basis of data from the NFZ. For the incidence of T2DM in Polish schizophrenic patients with reference to all patients with schizophrenia in the years 2010–2017, the relative risk (RR) with 95% confidence interval (95% CI) was calculated (12). RR compares the risk of a health event among one group with the risk among another group. This calculation is the result of dividing the risk (occurrence of schizophrenia) in group 1 by the risk in group 2. These two groups differ in terms of the incidence of T2DM (e.g. T2DM patient population versus the entire Polish population).  Page 8 section Statistical analyses |
